# Bioactive Peptides and Dietary Polyphenols: Two Sides of the Same Coin

**DOI:** 10.3390/molecules25153443

**Published:** 2020-07-29

**Authors:** Rosa Pérez-Gregorio, Susana Soares, Nuno Mateus, Victor de Freitas

**Affiliations:** REQUIMTE/LAQV, Faculdade de Ciências da Universidade do Porto, Rua do Campo Alegre 689, 4169-007 Porto, Portugal; nbmateus@fc.up.pt (N.M.); vfreitas@fc.up.pt (V.d.F.)

**Keywords:** absorption, health benefits, protein-polyphenol interaction, proline-rich proteins

## Abstract

The call for health-promoting nutraceuticals and functional foods containing bioactive compounds is growing. Among the great diversity of functional phytochemicals, polyphenols and, more recently, bioactive peptides have stood out as functional compounds. The amount of an ingested nutrient able to reach the bloodstream and exert the biological activity is a critical factor, and is affected by several factors, such as food components and food processing. This can lead to unclaimed interactions and/or reactions between bioactive compounds, which is particularly important for these bioactive compounds, since some polyphenols are widely known for their ability to interact and/or precipitate proteins/peptides. This review focuses on this important topic, addressing how these interactions could affect molecules digestion, absorption, metabolism and (biological)function. At the end, it is evidenced that further research is needed to understand the true effect of polyphenol-bioactive peptide interactions on overall health outcomes.

## 1. Introduction

Changes in diet and lifestyle adopted within the last decades in industrialized countries have promoted the growing burden of non-communicable diseases—mainly cardiovascular diseases, cancer and metabolic diseases, such as diabetes and obesity. Globally, the nutrition patterns go to an increase in the intake of fat, meat and ultra-processed foods while fruits, vegetables and wholegrains have been declining. In this framework, nutritional recommendations advocate for an increase in fruit and vegetable consumption. The health-benefits associated with these recommendations emanate from the bioactive nutritive molecules (mainly micronutrients), as well as from the non-nutritive bioactive compounds (polyphenols and bioactive peptides) from plant-derived foods.

Indeed, the call for health-promoting nutraceuticals and functional foods containing bioactive compounds is growing. Among the great diversity of functional phytochemicals, polyphenols and, more recently, bioactive peptides have stood out as functional compounds. Indeed, a lot of research has been made in order to establish priority actions to control the incidence of non-communicable diseases. One of these priority issues relies on research focused on the interaction between food components. The bioavailability or the amount of an ingested available-nutrient able to reach the bloodstream and exert that the biological activity is clearly more relevant than the total content from the whole food. In this way, the entire diet must be considered, and even the way food components are ingested or processed. Food processing, commonly used with different purposes (e.g., to kill microorganisms, enhance tastiness and increase shelf-life) can lead to unclaimed interactions and/or reactions between bioactive compounds.

This issue is particularly important for polyphenols and bioactive peptides, because some polyphenols are widely known for their ability to interact and/or precipitate proteins/peptides.

Although the biological fate of peptide-polyphenol complexes in vivo is unclear, the co-administered dietary compounds may influence the biological activity and bioavailability of both polyphenols and bioactive peptides. This review summarizes the relevance of the research of polyphenol–bioactive peptide interactions in the analysis of the healthy effect of a diet rich in fruits and vegetables. The first section of this review will be focused in the description of these particular plant-based bioactive compounds, then the possible effects of polyphenol-peptide interactions in their bioaccessibility, bioavailability and bioactivities from intake to health effects will be summarized. At the end, the food matrix effect will be also considered.

## 2. Bioactive Compounds from the Plant Kingdom

Among the great diversity of functional phytochemicals, polyphenols have been the most widely studied. Important bioactivities have been extensively demonstrated and linked to their consumption such as antioxidant, antihypertensive, hypocholesterolemic and immunostimulating properties. More recently, bioactive peptides from vegetal origin also stand out as functional compounds.

The therapeutic use of both polyphenols and bioactive peptides through diet is still under study, because of concerns about bioaccessibility, pharmacokinetics and bioavailability that could influence the real use of these compounds by the organism. In the specific case of bioactive peptides, there is the raised gastro-intestinal digestibility, inconstant absorption rate, the missing links in the study of mechanisms of action and the lack of well-designed clinical trials to afford the confirmation for potential health claims for further research. Furthermore, the way food components interact with each other affects the physiological processes, such as digestion and metabolism, which ultimately affect their bioactivities. This is particularly important for these classes of compounds. Indeed, the ability of polyphenols to bind to proteins and peptides has been extensively demonstrated and impacts very different areas, such as sensory science, nutrition, and health [1]. In fact, polyphenols binding to salivary proteins is the main accepted mechanism for the astringency perception [2]. Polyphenols are also thought to interact with plasma proteins or digestive enzymes, as well as with dietary proteins, in the stomach and gut [3,4,5]. More recently, the ability of polyphenols to bind to immunogenic peptides has been studied, resulting in a decrease in peptides bioavailability and immunogenicity [6,7,8].

### 2.1. Polyphenols: Nature, Occurrence and Related Constraints

**Figure 1:**
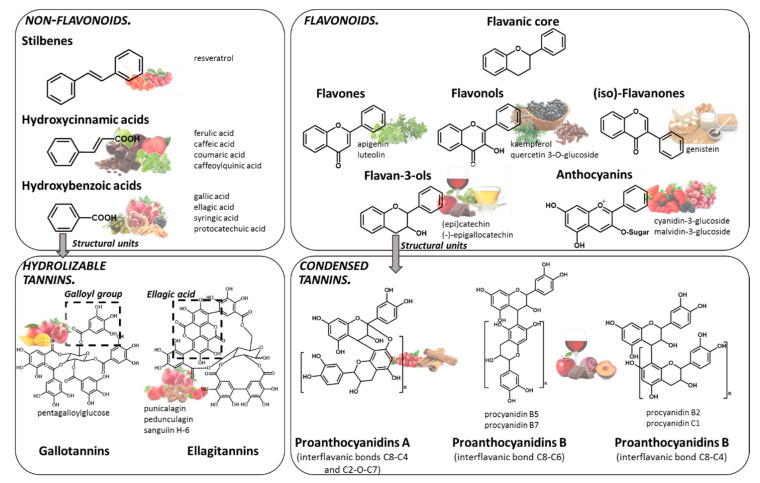
Chemical structure of the main families of polyphenols and some of the main food sources rich in each class: stilbenes (lingonberry, redcurrant), hydroxycinnamic acids (plums, coffee, chocolate, sweet cherry, broccoli, oregano, spearmint), hydroxybenzoic acids (pomegranate, blackberry, chestnut, walnut, olive), gallotannin (mango, pomegranate), ellagitannins (pomegranate, strawberry, pecan, raspberry, cloudberry), flavones (marjoram, oregano, sage), flavonols (black chokeberry, cloves, cumin), (iso)-flavanones (soy-based products), flavan-3-ols (tea, apples, wine, chocolate), anthocyanins (red raspberry, blackcurrant, red grapes, strawberries), proanthocyanidins (wine, plums, cranberries, chocolate, apple, cinnamon).

Polyphenols are compounds resulting from the secondary metabolism of plants, with more than 8000 structures currently known [9]. The general structure of polyphenols comprises at least a benzene ring, with a minimum of one hydroxyl group attached to it. Indeed, they can be classified into different groups according to structural differences, such as the number of phenol rings in the molecule, or the substitution groups of the phenol rings. Although several classifications have been made, the most accepted divides polyphenols into two main families: flavonoids and non-flavonoids (Figure 1).

Among the most common dietary non-flavonoids are the phenolic acids, namely hydroxycinnamic and hydroxybenzoic acids, their conjugated derivatives, and stilbenes. The members of the flavonoids’ family have a typical C6-C3-C6 flavanic core (Figure 1), and are the major polyphenols found widely spread through the plant kingdom and are, therefore, abundant in the human diet. The main sub-classes of the dietary flavonoids are flavones, anthocyanins, flavonols, (iso)-flavanones and flavan-3-ols (Figure 1). Among polyphenols, tannins represent a special group that have the ability to interact and precipitate proteins [10]. Tannins are divided into hydrolyzable tannins and condensed tannins. Their building blocks are based on monosaccharide esters derived from gallic acid or units of flavan-3-ols, respectively. Tannins are particularly important, since they can attain very high molecular weight and complex structures.

Understanding the intake of polyphenols needed to yield healthy outcomes goes through the deep knowledge of polyphenols nature and food distribution. This creates the first challenge, since the isolation, characterization and structural identification of the major polyphenols in dietary sources is still facing many difficulties. The difficulty to extract completely the polyphenols linked to fiber, the wide variety of chemical and highly complex structures and the different proposed methods of analysis clearly difficult to estimate their real content in foods. Furthermore, polyphenols content in foods depend on several intrinsic and extrinsic factors, such as variety, environmental conditions, agronomic factors, etc. [11,12]. Moreover, the industrial or culinary processing affect polyphenol content and bioavailability but could also induce structural changes in cell tissues, affecting their interactions with other food components.

This great diversity of polyphenol content, as well as the analytical methods used to characterize them, lead to some heterogeneity in total polyphenol amount in a diversity of foods after compilation of the literature data.

Diverse analytical methods can be used to encompass the whole variety of plant polyphenols, both in a quantitative as well as qualitative way. Colorimetric assays for global quantification or analysis of specific groups of polyphenols have been used (e.g., Folin-Ciocalteu, phloroglucinol and absorbance measurements at 520 nm) to determine the total polyphenol, total procyanidin and anthocyanins amount in foods, respectively [13,14]. For these methods, the presence of interferences could lead to over or underestimation of the polyphenol content on the assay. In fact, the most accurate method to characterize food polyphenols is the analysis of all individual analytes by high-performance liquid chromatography (HPLC). Nonetheless, the tremendous number of polyphenols and the lack of standards require different HPLC methods, as well as the calculation basis of each individual compound expressed in a model standard. Some different databases summarizing the polyphenols composition in food sources have been designed, such as the USDA database and Phenol-Explorer [15,16]. According to these databases, and to the general literature published, the richest sources of polyphenols can be summarized as follows [9]: spices and herbs have high amounts of flavanones and hydroxycinnamic acids; cocoa and tea leaves contain mainly procyanidins; a number of berries and red fruits have a high content of anthocyanins; pecan nut, hazelnut and almond are rich in proanthocyanidins, flaxseed is rich in lignan secoisolariciresinol, walnut and chestnut are rich in ellagitannins and roasted soybean and soy flour are rich in isoflavones. Other polyphenol-rich vegetables are black and green olives, artichoke heads, garlic, onion, spinach, endive, broccoli, potato, asparagus, lettuce, endive (escarole), carrot and wholegrain cereals. Furthermore, plant-based beverages also contain great content on polyphenols such as coffee (rich in chlorogenic acids), tea (rich in catechins, proanthocyanidins and theaflavins), beer (containing hydroxycinamic acids and proanthocyanidins) and red wine (containing proanthocyanidins, anthocyanins and hydroxycinnamic acids).

In this way, depending on diet, people can have an average intake of a gram of polyphenols per day. In the US, the average daily intake only for proanthocyanidins has been estimated to be 189 mg/day [17]. Recently, an extensive review on the dietary intake of polyphenols settled in 900 mg/day the polyphenol consumption for the overall population [18]. In both studies, the main food sources of polyphenols were represented by red wine, tea, coffee, fruits and vegetables.

**Figure 2:**
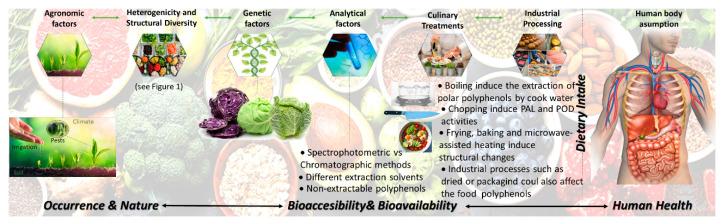
Factors affecting the relationship between dietary intake of polyphenols and healthy outcomes.

Despite the widespread data on the characterization and quantification of the food content on polyphenols, as referred to previously, during technological and culinary treatments, important chemical and biochemical reactions occur in the raw plant tissues. Such reactions may have an impact on the polyphenols structure, resulting in the degradation and/or the formation of new compounds [19,20]. The changes induced by these extrinsic factors joined with the lack in the understanding of their bioavailability clearly difficult the understanding of the optimum polyphenols intake to achieve specific health outcomes, as summarized in Figure 2. These effects should be also considered and may be introduced into databases to be further used as a reference. In addition, the total content of polyphenols of a food is uncertain to entirely illustrate any related health effects given the great dissimilarities in the bioavailability, bioaccesibility and bioactivity of isolated polyphenols, which have to be considered. The limitations of the current data should not be ignored.

A systematic review of the literature from the last 10 years about polyphenol intake and health outcomes, summarized that overall flavonoids and specific subclasses (e.g., proanthocyanidins), but not total polyphenols, have been apparently linked to a low risk of non-communicable diseases [18].

Overall, there has been an increased awareness in the use of polyphenols as modulators of a quantity of non-communicable diseases, but the real mechanisms of action in the human body are still the focus of a lot of research. Some of the most important constraints are due to the nature, occurrence, digestion, metabolism, interactions with other food components and with cell receptors, human body enzymes or gut microbiota. Some of these constraints will be discussed in this review.

### 2.2. Bioactive Peptides from the Plant Kingdom

Bioactive peptides are peptides that have biological activities in most living organisms, including humans, animals and plants. Over the years, a vast number of in vitro and in vivo studies with animal trials have pointed that in humans these peptides are tangled in a wide range of important bioactivities, such as antihypertensive, hypocholesterolemic, anti-tumoral, anti-proliferative, antimicrobial, osteoprotective, antioxidant, immunomodulatory, opiate and anti-inflammatory properties. Table 1 summarizes the major sources of bioactive peptides studied in relationship with the biological activities described.

The potential of peptides for being used as bioactive compounds could be anticipated. Certain amino acid sequences control and direct cellular function and manage most intercellular communication. Some structural features in peptide sequencing have been highlighted as affecting their functionality: in general, proline-rich peptides (PRPs) stand out as the most active, while cysteine is the less abundant amino acid within bioactive peptides [44]. The importance of proline residues in a peptide sequence relies on conferring resistance to cleavage by digestive enzymes, which is also due to its high hydrophobicity. Furthermore, while aromatic amino acids (tyrosine and phenylalanine) and arginine are usually cleaved at C-terminal, the most frequent N terminal residues are leucine, valine, isoleucine and tyrosine.

In addition to peptide sequencing, the bioactivities may also depend on the chain length. For example, soybean-derived peptides from 13 kDa to 1 kDa displayed antioxidant, as well as improvement of muscle glucose uptake activities, while larger peptides have been related with hypocholesterolemic activity [45]. In this context, peptides ranging from 200 to 3000 Da have been described as stimulators of LDL receptor transcription, thus prompting a decrease in the levels of circulating LDL [46]. In addition, some peptides with less than 10 residues, such as the X-Met-Leu-Pro-Ser-Tyr-Ser-Pro-Tyr obtained by de-fatted soy protein, the Glu-Gln-Arg-Pro-Arg, isolated from heat stabilized de-fatted rice bran or oryzatensin, Gly-Tyr-Pro-Met-Tyr-Pro-Leu-Pro-Arg, obtained from rice soluble protein have shown anti-inflammatory, antiproliferative and antimicrobial activities, respectively.

The smaller size of peptides has been related to a higher absorption rate, resulting in higher bioactivities when compared with bigger peptides. For example, antimicrobial peptides from plants usually have molecular weights between 2–10 kDa, with mainly positive charges, and containing cysteine residues for stability. Recently, a comprehensive revision provided the state-of-the-art on the correlation between the bioactive peptides structure and their related biological properties [44].

Bioactive peptides (3–20 amino acid residues) may be embedded in the amino acid sequence of a protein and released after precursor degradation. The precursor proteins are usually pharmaceutically inactive, or exert specific functions in their native organisms, whereas their hydrolysis products could represent bioactive peptides with functionalities completely different from native proteins and are usually referred as “cryptides”. In plants, the precursor proteins and/or the bioactive peptides are involved in the defense response, as well as in signaling pathways and the development regulation [41].

These precursor proteins can occur inherently in foods or can be supplemented to foods. Then, the bioactive peptides can be naturally released during protein digestion in vivo upon oral intake throughout the gastrointestinal tract, and they can exert bioactivity in the small intestine and large bowel. The bioactive peptides described as resulting from gut microbiota metabolism exerting their bioactivities at the large bowel are limited to those protein precursors able to reach the colon. Upon their release from precursor proteins, bioactive peptides must persist active and unbroken during gastrointestinal digestion and absorption to reach the bloodstream, building their biological functions. In fact, once in the human body, all peptides must pass by several barriers that can disable them and subsequently lose their biological action.

Nonetheless, bioactive peptides can also occur in food matrices by using biotechnology approaches, namely microbial fermentation, being plentifully present in fermented dairy products and by the production of protein hydrolysates using enzymes. The oral intake of these type of food matrices (rich in bioactive peptides) faces some other challenges, especially regarding taste. Bitter taste has been associated with both bioactive peptides and proteins, which may influence consumer satisfactoriness [47]. Several studies have highlighted structural features with a propensity towards bitterness, such as increasing molecular weight, the presence of certain amino acid sequences, the degree of electrical charge and, interestingly, also the presence of hydrophobic amino acids at the C-terminal (reviewed in [48]). Traditionally, the decrease levels of these bitter-tasting peptides have been performed to improve the organoleptic properties. One of the events comprises the further hydrolysis of the compound (the food fermented product or the protein hydrolysate) by enzymes, to diminish the amount of any bitter-tasting peptides [49,50]. While this approach could be effective to reduce bitterness, this procedure can potentially abolish by “mistake” the very bioactive stuffs that made the food matrix valuable in the initial place.

The substitute choice has been to “screen out” bitter peptides while the lack of a widespread structure-activity relationship in taste research of bioactive peptides further hinders an effective global application. Moreover, these data must be linked to the potential bioactivity of the screened peptides, adding an unbearable amount of time and expense to such approach. So, it could be challenging to ally richness in bioactive peptides with consumer taste acceptability for a food product.

In summary, in the human body, bioactive peptides could arise mainly from three different ways: (i) natural occurrence in food sources; (ii) from dietary proteins by natural (digestion) or artificial proteolysis (e.g., from fermentation, enzyme hydrolysis); or (iii) by the action of the microbiome and cell metabolism.

Food-derived bioactive proteins and peptides from animal source have been more widely studied mainly haemoglobin, immunoglobulins, serum albumin, salivary and milk-derived bioactive peptides. These peptides arise mainly from milk, meat and egg. At present, milk proteins are considered the most important source of animal-based bioactive peptides. All these peptides have been extensively revised, so they will not be focused on within this revision [51,52,53,54,55,56,57].

On the other hand, the interest on plant-based bioactive peptides is more recent. These plant-based peptides present important advantages regarding animal peptides. Moreover, allied to bioactive peptides, plants present a huge potential having other nutraceutical compounds, such as polyphenols. In the last years, research in this area has emerged, as recently reviewed [41]. Here, the current knowledge on these plant-based bioactive peptides will be focused first and then the possible interactions with polyphenols will be further deepen. Some cardiovascular benefits, derived from reduction of blood pressure and hypercholesterolemia or antithrombotic effects have been described after the consumption of bioactive peptides from plant origin, but they have also been claimed as antioxidants, enhancers of trace mineral absorption, opioids and with immunomodulatory activities.

Vegetables and fruits are usually consumed, and agro-industrial wastes comprise a cheap, huge and eco-friendly source of proteins [58]. So, the production of bioactive peptides from these by-products as a possible way to reduce waste and recover value-added substances appropriate for functional food production and nutraceuticals is a significant advantage.

Cereals and legumes stand out as the most explored vegetables as sources for bioactive peptides. Nonetheless, research on other vegetables has been made as well, including pseudocereals, spices, seeds, alfalfa and common edible plants (spinach, cocoa beans). Among cereals, wheat, soy, maize, sorghum and rice have been widely studied.

Lunasin is an innovative 43-amino-acid bioactive peptide that naturally occurs in several plants. The carboxyl end of lunasin comprises a typical cell adhesion motif with nine aspartic acid residues. Lunasin has been suggested to have significant health benefits, namely hypocholesterolemic, antioxidant, anticancer and anti-inflammatory activities, and was firstly identified and isolated from soybean [59]. Later, it has been also reported to occur in wheat [60], barley [61], oat [62], rye [63], amaranth seed [64] and, more recently, in quinoa [65].

Additionally, potato protein isolates have been known to produce angiotensin I-converting enzyme (ACE) inhibitory peptides by autolysis. ACE inhibitory peptides are widely used to control hypertension. Mäkinen and colleagues found that the potato protein-derived ACE inhibitory peptides were rich in glutamic acid, glycine, leucine and serine residues, although they were not able to identify the peptides sequences [66]. The existence of hydrophobic groups is proposed to play a primary role in the ACE inhibitory function of protein hydrolysates and peptides.

An important class of bioactive peptides naturally occurring in plants are the cyclotides. Structurally, they are disulfide-rich peptides with a head-to-tail cyclized backbone and knotted arrangement of three-disulfide bonds. Several biological activities have been linked to cyclotides, namely protease inhibition, antimicrobial, insecticidal, cytotoxic, anti-human immunodeficiency virus and hormonelike activities [67]. These compounds are actually obtained from aqueous plant extracts and have been applied in the form of infusion or for topic skin use [68].

Some other peptides naturally present in plants are also bioactive, such as the defensins. They have been shown, in addition to their antimicrobial activity, to confer cytotoxicity and anticancer activity against many types of cancers. Defensins, one of the earliest reported antimicrobial peptides, Ligatoxin B, lectin and other small proteins from mistletoe, coccinin from runner beans, are those reported to have cytotoxic activities [69].

Among cereal proteins, barley or walnuts also contain naturally bioactive peptides, but the vast majority of cereal bioactive peptides come from the sourdough fermentation [41]. The effect of microbial population during sourdough fermentation is being studied. It seems that the Lactobacillus strains produce fermentation products with higher antioxidant activity than control antioxidants due to the release of bioactive peptides from gliadins ranging from 8–57 amino acid residues [70]. Soybean proteins, β-conglycinin and glycinin, have been also recently studied as a good source of bioactive peptides by means of gastric, pancreatic enzyme, as well as gut microbiota digestion [71].

The release of bioactive peptides from soybean seeds and soy milk protein samples was observed after mimicking the in vitro gastrointestinal digestion and then analyzed by LC-MS/MS for peptide sequencing [72]. The peptides sequenced were then searched on databases to further match with already described bioactive peptides. The results revealed that soybean proteins experienced a high hydrolysis during gastrointestinal digestion, releasing a large number of bioactive peptides with antimicrobial properties.

Additionally, quinoa peptides released during gastrointestinal digestion showed potent in vitro anti-diabetic properties [73].

The hydrolysis by digestive enzymes is also frequently performed in vitro. In fact, a large number of bioactive peptides are also released upon the action of trypsin, pepsin and chymotrypsin digestive enzymes. A trypsin digest of soy protein extract presented the soymetide peptide (MITLAIPVNKPGR) as an active component, and was able to stimulate phagocytosis in vitro [74]. The peptide name was due to the crucial methionine residue at the amino terminus, essential for its activity.

Canola, soybean, sunflower and peas have also been described as a good source of bioactive peptides in the treatment of hypertension [75,76,77,78].

Canola protein hydrolysates obtained after 4 h hydrolysis using different proteases (e.g., alcalase, chymotrypsin, pepsin, trypsin and pancreatin) were able to reduce blood pressure in spontaneously hypertensive rats [79]. Several antioxidant peptides have been obtained resulting from chickpea seed proteins hydrolysis with pepsin and pancreatin [80]. Most of these peptides encompass the antioxidant amino acid histidine, and belong to legumin fragments, the main chickpea seed protein. Rice bran protein has also been hydrolyzed using trypsin and a novel antioxidant and antihypertension peptide was found, named as F2-a [81]. Additionally, walnut protein hydrolysates, which are mainly the ones obtained from pepsin proteolysis, showed antioxidant activities inhibiting lipid peroxidation, quenching hydroxyl radicals and exhibiting reducing power [82]. Walnut protein hydrolysates are also able to chelate ions such as ferrous ion.

Rapeseed protein isolate, usually used in the formulation of livestock feed, is an innocuous innovative food ingredient (European Food Safety Authority, 2013) and has been recently recognized as a good source of bioactive peptides [83]. In fact, rapeseed protein isolate has been determined as a good source of bioactive peptides with antioxidant abilities and ACE inhibitory capacity [66,79,84]. Furthermore, the lactic acid fermentation also produces ACE inhibitory and antioxidant peptides from rapeseed [83].

## 3. Bioactive Peptides and Polyphenols

When reviewing plant-based bioactive peptides several aspects can be considered, as described in the previous topic (e.g., ranging from spontaneous production or preparation to processing effects, identification of such peptides in protein hydrolysates, structure/bioactivity relationship, the need for evaluating bioavailability, etc.). Although these aspects are also considered here, as well as in most reviews, one often neglected aspect is the interaction with other plant-based compounds such as polyphenols.

Moreover, since different foodstuffs have diverse bioactive compounds with various biological capacities, specific foods, when consumed together, may produce interactions, which could lead to synergistic or inhibitory effects that, in turn, have a greater effect on physiological health than when consumed alone.

Within this framework, polyphenol compounds have a critical importance due to their well-known ability to bind to proteins and peptides. This ability has been extensively studied, mainly applied to food quality. Indeed, this property has been associated with organoleptic properties of foodstuffs, such as flavor and taste attributes (astringency and bitterness). Furthermore, among food macronutrients, polyphenols have also been described as being able to interact with carbohydrates [85] and with lipids [86,87], as well. In fact, polyphenols have complex mechanisms being able to interact simultaneously or by competition with mixtures of carbohydrates and proteins [85]. From a mechanistic point of view, non-covalent binding between polyphenols and macronutrients is mainly due to electrostatic, van der Waals forces, hydrogen bonding, hydrophobic effect but covalent binding has been also described [88]. The nature as well as the mechanisms of the interaction depend on different environmental factors, such as ionic strength, pH or compounds concentration, and are also influenced by structural features, namely molecules polarity, steric conformation and size.

### 3.1. Interaction at a Molecular Level

Focusing on the polyphenol’s interaction with the precursor proteins or with the bioactive peptides, several implications in molecules digestion, absorption, metabolism and function could be anticipated due to these interactions.

To date, most of polyphenol-protein/peptide interactions have usually been studied in vitro, with isolated purified compounds or standards, quite far from what occurs in food matrix and during food intake. Different techniques have been used in the characterization of polyphenol-protein/peptide complexes, such as chromatography (HPLC) [89], electrospray mass spectrometry (ESI-MS) [90,91,92,93], capillary electrophoresis (SDS-PAGE) [94], nuclear magnetic resonance spectroscopy (STD-NMR) [90,95,96,97], nephelometry and turbidimetry [98], small-angle X-ray scattering (SAXS) [99], isothermal titration calorimetry (ITC) [100], dynamic light scattering (DLS) [99,101] and fluorescence spectrophotometry [102,103]. Binding affinities, structure/activity relationship, binding epitopes and environmental conditions affecting these interactions have also been studied.

The extensive literature on this topic allows to have some general structure-binding guidelines, although it is important to underline that particular structural changes could have also prominent effects on these interactions. Polyphenols have a higher ability to bind to PRPs, although not specifically to the proline residues. This higher ability is generally described to be due to a more open and extended conformation of the peptidic chain, usually imposed by the proline residues. Keeping in mind that bioactive peptides are usually rich in proline residues, and considering the roles of polyphenols in controlling proline-rich protein gene expression, a new research field is open for nutritionists. In fact, this higher affinity toward PRPs occurs for both biological proteins (e.g., salivary PRPs), as well as for food proteins (e.g., gliadin).

Polyphenols with a higher molecular weight, as well as with a higher number of hydroxyl or galloyl groups, present a higher number of sites for interaction with proteins.

The polyphenol-protein/peptide interactions could be reversible or irreversible [5]. As referred to previously, these interactions can occur through non-covalent bonds, which usually results in reversible interactions. However, the extent of these interactions could achieve a large network of aggregates that eventually become insoluble leading to the irreversible precipitation of polyphenol-protein/peptide complexes. More often, the irreversible interactions occur through covalent bonds, promoted by the ability of polyphenols to produce quinone radical.

Various studies have also recognized the association between flavonoids, phenolic acids and quinones with plant-based proteins such as soybean isolates [104], coffee storage protein [105], white bean albumin and globulin [106], proteins in honey [107] and rice glutelin [108]. The interaction with polyphenols has been also identified in plant-based protein hydrolysates, namely rapeseed protein hydrolysates and more extensively reported with gluten wheat protein [109,110].

Brudzynsky and colleagues showed that the redox reactions that occur during honey storage between protein amino acids, reducing sugars and polyphenols form high molecular weight protein insoluble aggregates [107].

Swieca and co-workers found that the fortification of wheat bread with onion skin polyphenols (containing mainly quercetin) significantly reduced the protein digestibility, and insoluble complexes were observed [109]. A vast amount of the literature is devoted to the interaction between wheat proteins, mainly gluten and derived proteins, such as gliadins and glutenins, and polyphenols, especially from a food quality point of view [110,111,112]. These interactions are known to affect bread structure and properties, the baking process and dough development. Moreover, gluten and derived proteins and peptides have a particular interest, because they are PRPs, being expected to have a significant interaction with polyphenols. This raised the hypothesis of using polyphenols to modulate celiac disease, since these peptides are a key factor in this disease. The effect in the immunogenicity and absorption of these peptides by epigallocatechin gallate (EGCG) has been already studied after the characterization of the molecular interaction [7,113]. However, further studies are required with other polyphenols structures and peptides. In fact, few studies have been focused in the effect of polyphenols interaction in mediating proline-rich protein-related diseases, such as celiac disease.

In summary, depending on the reversibility and/or (in)solubility of the referred protein-polyphenol aggregates, several aspects could be impaired, namely protein bioavailability and (precursor) protein digestibility, which as a side effect that could affect the formation or the functionality of the bioactive peptides upon the gastrointestinal digestion.

**Figure 3:**
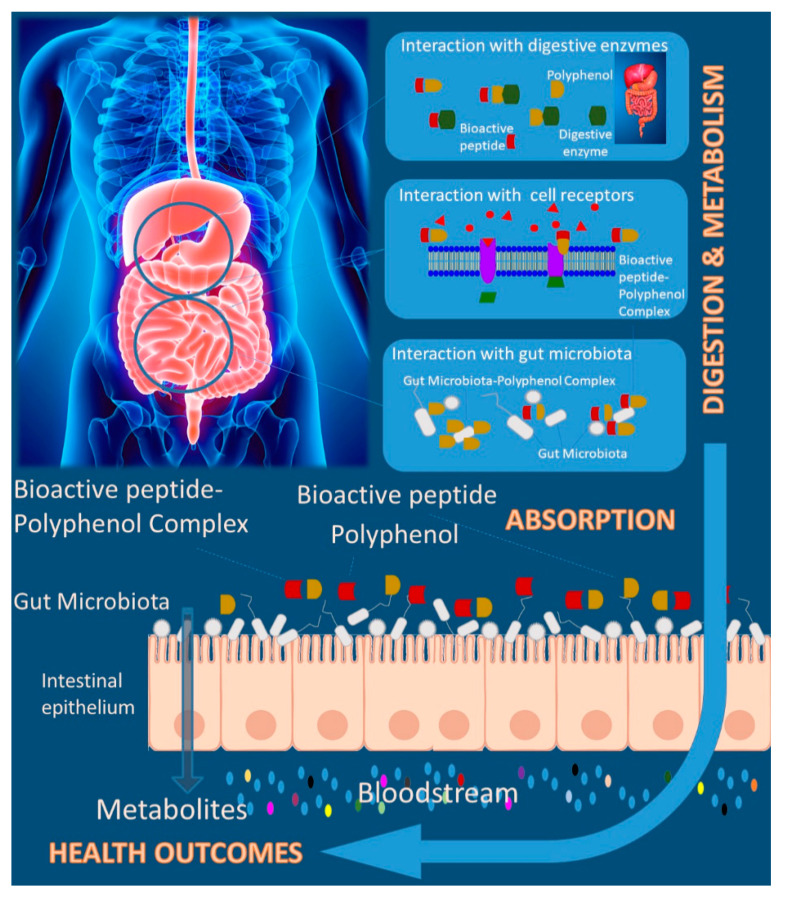
Polyphenol-bioactive peptide interaction and health-related effects.

While many features of the polyphenol–protein interactions are well understood, the in vivo implications are poorly explored, and important questions remain unanswered. Figure 3 summarizes the main effects promoted by bioactive peptides-polyphenols interactions in their biological activities. The potentially effect of these interactions under biological systems claims for going deeper on the design of complex models and discover the underlined mechanisms under in vivo and human studies. While, from one side, polyphenols may be beneficial in modulating bioactive peptides resistance to further hydrolysis (soluble complexes) and thereby increasing the efficacy and healthy effects, from the other side they could precipitate the bioactive peptides (insoluble complexes). In addition, these properties were known to have been associated with the influence of dietary polyphenols on bioactive peptides effectiveness as a result of dietary polyphenol-bioactive peptide interactions. However, no data is available on the influence of polyphenols on the study of plant bioactive peptides [114].

Due to the importance of both these dietary bioactive compounds to human health and disease, there is a dynamic interest on the study of their bioeffects. For instance, a comprehensive understanding of the interactions must be achieved by exploring additional types of polyphenol structures. In fact, the use of polyphenols from food wastes as dietary supplements or functional foods that has risen in recent years in a circular economy perspective, claims for the characterization of the new dietary polyphenols. The nature of polyphenol-protein interactions routinely assessed in vitro should also be evaluated at the gastrointestinal environment conditions, such as temperature, pH or the presence of bile salts and carbon dioxide to understand their stability. Hence, (1) the effect of peptide or protein flexibility and polyphenol structure to modulate the interaction; (2) competition mechanisms and structural features derived from food matrix; (3) the stability of these interactions under biological conditions, and, consequently, the interplay between hydrogen bonding and hydrophobic interactions; (4) the real effect in the employment by the human organism urges further and deeper research. However, the structural complexity of both families of compounds, and the innumerable factors that affect their interactions, are a long-term challenge for the field of food science. It is likely that polyphenol–protein interactions will remain an active research area for many more years.

### 3.2. Bioactive Peptides and Dietary Polyphenols: Gastrointestinal Digestion

Bioactive peptides bioavailability after oral administration is one of the main concerns in the study of the relationship between intake and health effects manifestation. During digestion, proteolytic gastrointestinal enzymes can release peptides from food precursor proteins with bioactive properties. In addition to gastric and small intestinal digestion, peptides can be further digested into small peptides and amino acids by endopeptidases from brush border membranes. The degradation pattern depends on the peptide’s length. Indeed, di- and tripeptides can be absorbed intact, whereas higher peptides are extracellularly hydrolyzed at the brush border membrane of the intestinal epithelium. In this framework, proteins digestibility is the main constraint factor since bioactive peptides must be “released” from the precursor proteins, and they should remain intact until reaching the target organ.

Protein digestion begins in the stomach at an acidic pH via pepsins. There are several luminal, circulating, secreted, intracellular, intramembrane or pericellular enzymes affecting the process. Indeed, after pepsin cleavage, peptides will be further hydrolyzed by pancreatic proteases, α-chymotrypsin, trypsin, elastase and carboxypeptidase A and B at duodenum under an alkaline environment [115]. Most oligopeptides and some free amino acids are produced in the lumen of the small intestine by the peptidases in the intestinal villi. The most common peptidases from villi are endopeptidases, dipeptidases, aminopeptidases and carboxypeptidases.

Usually, the digestion process is studied in vitro by using isolated standards or extracts, but the interaction between dietary components and the related consequences in compounds bioactivity remain unexplored. As mentioned, polyphenols have the overall ability to bind to proteins, including digestive proteases, such as pepsin and trypsin, as displayed in Figure 3. This has been thoroughly revised previously [4,116]. The binding of polyphenols to proteases can have various effects: inhibition, as demonstrated in most of the studies, activation, or no effect at all. Pepsin activity has been shown to be inhibited by several flavonoids such as epigallocatechin gallate (EGCG) [117], one of the major tea polyphenols, by catechin [118] and by baicalein [119]. Trypsin has been shown to be inhibited by condensed tannins with a clear relationship between the degree of polymerization of procyanidins and the enzymatic inhibition observed [120]. Trypsin has been also inhibited by different phenolic acids [121]. EGCG was also found to inhibit trypsin [118,122]. In fact, EGCG also inhibited pancreatic lipase and phospholipase A2. So, two outcomes could be expected from these interactions: from one side, the formation of bioactive peptides from precursor proteins by action of these proteases can be compromised, but on the other hand, this could protect bioactive peptides from early degradation.

The greatest threat to bioactive peptides occurs in the lumen of the small intestine, which contains pancreatic proteases and proteases from the mucosal cells, which are regularly released from the villi and could further hydrolyzed them. The second major enzymatic barrier is the brush border membrane of the epithelial cells, which contains at least 15 highly active peptidases. Indeed, according to Woodley [123], an orally administered protein could encounter over 40 different enzymes during its passage through the small intestine. Furthermore, over 60 lysosomal peptidases have been estimated to be able to degrade endocytosed peptides.

However, some specific peptide motifs are partially resistant. PRPs as well as hydroxyproline residues have been widely recognized as able to resist the gastrointestinal hydrolysis by digestive enzymes. The ability to resist the degradation during in vitro digestion drives to an increase of the biological action of some peptides while decreasing it in others [124]. Mimicking the physiological digestion in vitro is a very useful tool in the analysis of bioactive peptide stability, biological activation and action mechanism of peptides with already known in vivo bioactivity. However, further research is needed to go further and understand the digestion process, as well as to improve the knowledge of the enzymatic population and activity from all compartments of gastrointestinal tract and cleavage patterns is crucial in the study of peptides bioactivity.

Thus, changes in the bioactive peptide formation, bioavailability and functionality could arise from all these complex interactions at the gastrointestinal environment.

It is also possible that undigested and/or non-absorbed peptides reach the large intestine, where they could be metabolized by gut microbiota. Polyphenols’ ability to bind to and modulate gut microbiota has been highlighted recently [125]. In fact, this relation has been referred as a two-way relationship “polyphenols ↔ microbiota”, since dietary polyphenols modulate the colonic microbial population composition or activity and this population also metabolizes differently the several polyphenols. So, this has a double outcome for bioactive peptides. Since polyphenols affect the microbial species and strains present [126,127], the metabolization of bioactive peptides is supposed to be different. Furthermore, since polyphenols are also metabolized [128], new polyphenol metabolites appear and could interact with the bioactive peptides.

As referred to above, despite polyphenols occur in foods mainly as esters or glycosides and polymers, they are digested by system enzymes or intestinal microbiota prior to be absorbed and used by the organism. According to previous studies, 48% of ingested polyphenols are estimated to be degraded in the small intestine and 42% in the large intestine, while 10% are undigested [129].

Among the great variety of polyphenols structures, the lower size of free phenolic acids facilitates their absorption but their conjugation with glucuronide acid also promote their rapid absorption. The lipophilic properties of flavonoid aglycones allows them to cross biological membranes, being partially absorbed in the native form. Indeed, in vivo studies with mice model found phloretin, in both conjugated (90%) and non-conjugated (10%) forms in plasma [130]. Additionally, glycoside flavonoids are linked to monosaccharides, such as glucose, rhamnose or galactose or disaccharides like rutin or neohesperidin. The digestion of this group of polyphenols starts at the oral cavity by means of β-glycosidase, after that, they are further degraded in the stomach under a chemical hydrolysis, due to the low pH of this compartment [129]. Further reactions of glucuronidation, methylation, deglycosylation, sulphonation and hydroxylation of flavonoids occur in the small intestine and most polyphenol-derived metabolites are adsorbed at duodenum [131].

Non-digested polyphenols such as procyanidin oligomers or esters of phenolic acids are partially degraded in the large intestine, where they are subjected to further transformation by gut microbiota [132]. Glycosilated polyphenols are also hydrolyzed by colon microbiota into aglycones, which are partially absorbed and transformed into various acids through the action of β-glucosidase, β-rhamnosidase and esterases. In contrast to phase I and II gastrointestinal enzymes, those of the gut microbiota are able to hydrolyze flavonoid chains into simple units. Furthermore, they can also perform hydrolysis, dehydroxylation, demethylation and decarboxylation. Moreover, the action of small intestinal brush-border membrane enzymes, such as phlorizin hydrolase, have already been studied [133]. A significant number of metabolites formed along the gastrointestinal tract may reach the bloodstream, being transformed in the liver and further secreted back into the gut with bile acids, where they are again catabolized and are either absorbed back or excreted via the feces. Indeed, the conjugation of isoflavones to glucuronide and sulphated derivatives occurs in the liver by UDP-glucuronosyltransferase and sulfotransferase enzymes, respectively. In vivo studies with animal models showed that the highest level of glucuronyl transferase activity was observed in the intestine, where quercetin glucuronides were formed and secreted back either to the gut lumen, or to the serosal side, thus entering an enterohepatic cycle. Then, these conjugates reach the liver, where they are further metabolized. In general, flavan-3-ols give rise to phenylvalerolactone and hydroxyphenylpropionic acids, flavones and flavanones render hydroxyphenylpropionic acids and flavonols degrade to hydroxyphenylacetic acids. Overall, polyphenols are far metabolized either in tissues from gastrointestinal tract, or by the gut microflora, for the re-excreted fraction in the bile and/or non-absorbed fraction. As referred to above, a large variety of compounds can thus be produced, depending on the structure of polyphenols, but in the end, they ultimately lead to phenylacetic, phenylpropionic, phenylvaleric and benzoic acids.

The metabolomics pattern of polyphenols leading to conjugated and methylated substances has been studied. However, most of the biological studies have only been carried with native polyphenols. Little is known on the bioactivities of polyphenol metabolites, due to the lack of commercial standards. Indeed, some controversial studies revealed that sulphate esters and glucuronides were shown to exert antioxidant effects, while other studies showed that glucuronidation of flavonoids reduces their biological activity. More such studies are needed to further investigate the digestion and metabolism of polyphenols and to properly evaluate the in vivo healthy outcomes.

### 3.3. Bioactive Peptides and Dietary Polyphenols: Absorption

Although the previously mentioned digestion and modifications are expected to occur, to a certain extent, it is also probable that a fraction of small-sized peptides or free amino acids can be directly absorbed in physiologically active amounts [134]. In vivo studies verified that peptides of different sizes, but with the carboxyl and amino terminal groups blocked, are able to pass through the intestinal epithelium barrier [135]. So, some peptides are able to reach the blood stream intact [124], although the efficient absorption of an intact peptide is the exception rather than the rule. Finally, the absorbed peptides could be further degraded at the liver, although the transit time is shorter and the enzyme activity lesser than in the gut.

The transport across intestinal epithelium can occur through one or more pathways [136]: PepT1-mediated permeation, paracellular transport via tight junctions, transcytosis and passive transcellular diffusion [135]. Bioactive peptides absorption via the PepT1-mediated permeation is complex, since it is a specific mechanism mediated by a receptor. PepT1 favorably binds peptides with short chains (i.e., dipeptides and tripeptides), high hydrophobicity and neutral charge. Oligopeptides can be absorbed by both receptor- or non-receptor-mediated endocytosis. In parallel, paracellular and transcellular routes could also allow the uptake of oligopeptides. The paracellular transport is a passive diffusion process mediated by intestinal tight junctions. The aqueous nature of this pathway favors the absorption of small hydrophilic solutes with net negative charge. Peptides can also reach the enterocytes by transcytosis, an energy-dependent transcellular transport, which favors the transport of peptides with long chains and high hydrophobicity. The energy that mediates this process is obtained from ATPase proteins from basolateral membrane and ion-specific transporters. For transcytosis, a link has been observed between the membrane and the peptides absorption. The passive transcellular diffusion comprises passive uptake of (preferably hydrophobic) peptides into cells, intracellular transport and basolateral secretion.

Overall, the absorption capacity of dietary peptides decreases as chain length increases at the same than when hydrophilicity increases. Hence, peptides and proteins displaying a molecular size greater than 30 Å have molecular hindrances to cross the intestinal membrane.

In the context of bioactive peptides absorption, polyphenols could influence in several ways and backwards. As referred to previously, the ability of polyphenols to bind directly to peptides could obviously influence the transport mechanisms and rate of absorption. On the other hand, polyphenols are also able to bind to cell receptors influencing the active transport across the epithelium [137], and are also described as modulators of the tight junction expression levels, relevant proteins in the paracellular transport [138].

On one side, the commonly deficient transport of peptides across biological membranes can be ascribed to their hydrophilic structure and molecular size, but on the other side, the binding affinity of polyphenols is clearly influenced by polarity and molecular weight. Indeed, the bigger is the peptide, the stronger is the binding ability [88]. The polarity of polyphenols and proteins influence the mechanism of interaction and the ability to form either soluble or insoluble protein/peptide-polyphenols complexes [88]. In this context, the possibility to explore the polyphenol-protein interaction in the absorption rate of bioactive peptides arises. Furthermore, research has shown that some dietary components are related with an increase in the oxidative stress in epithelial cells and further inflammation processes, which could degrade tight junction proteins and open the epithelial barrier. With both transcellular and paracellular pathways opened, large proteins can potentially be absorbed more rapidly and in greater amounts, having an impact in health status. Thus, not only the interaction between polyphenols and proteins, but also the ability of polyphenols to modulate the tight junction expression linked to the anti-inflammatory and antioxidant properties of both bioactive peptides, open a new perspective in the study of bioactive peptides absorption.

Overall, to highlight the region of the gastrointestinal tract that favors protein/peptide absorption is a key step in the design of oral delivery systems (supplements or bioactive-rich diets) for biologically active compounds. Certain factors affect molecules permeability through the intestinal epithelium barrier, such as intestinal barrier characteristics, structural and physicochemical properties of the molecule, intestinal fluid composition, pH and transport mechanism, and all of these factors are closely linked with the polyphenols ability to bind to peptides.

Regarding polyphenols, there is a paradox between the consumption of foodstuffs rich in polyphenols, the bioavailability observed after quantifying the excreted and absorbed bioactive compounds and the antioxidant capacity of the plasma following the intake. Polyphenols have been traditionally described as relatively poorly absorbed compounds. The circulating metabolites quantified are low and the absorption ranges from 0.3% to 43%, being the phenolic acids the most easily absorbed, whereas large molecular weight polyphenols, such as proanthocyanidins, are very poorly absorbed [139]. Low recovery in urine has been observed for some polyphenols, while the extent of biliary excretion of polyphenols has not been well explored in human studies. Few studies showed that polyphenols structure affect their urinary excretion, as well as intestinal absorption. The lowest recovery was achieved for tea theaflavins (0.0006%), followed by the flavonols quercetin or rutin (0.3–1.4%), tea catechins, soya isoflavones, flavanones from citrus fruits or anthocyanins from berries or red wine, whereas the highest urinary excretion ratio was obtained for caffeic acid (27%) [139]. In addition to the polyphenol structure, the main features affecting the process are glycosylation, esterification and molecular weight. The higher the molecular weight, the lower is the urinary recovery. Hence, since procyanidins usually occur as large polymers, they have been considered as the less absorbed along the gastrointestinal tract. However, the absorption patterns of polyphenols and gut microbiota biotransformations are a topic under study. Regarding the glycosylation and esterification patterns, most flavonoids appear glycosylated in dietary sources of polyphenols. Several studies verified the effect of glycosylation in the absorption through the gut barrier. Oliveira et al. [140] evaluated the role of isolated anthocyanins from purple fleshed sweet potatoes and grape skin in two glucose transporters (GLUT1 and GLUT3) under in vitro studies, mimicking the human gastric epithelial cells (MKN-28), and by using gold nanoparticles to silence these transporters. Results derived from this study clearly showed the relevance not only on the glycosylation pattern, but also if esterification in the transport mechanisms was mediated by membrane receptors. Furthermore, the transport activity of the main glucose transporter, the sodium-dependent glucose transporter, SGLT1, was also inhibited by green tea polyphenols influenced by galloyl residues [133]. Glycosylated onion flavonoids seem to be better absorbed than the relative aglycones. In vivo studies with animal models verified that plasma concentration of onion 3-O-β-glucoside of quercetin was significantly higher than quercetin after 4 h of oral administration. In contrast, rhamnosides of quercetin were poorly absorbed in the same conditions [141]. As referred to above, the deglycosylation pattern clearly affects the catabolism reactions by gastrointestinal enzymes, epithelium cell enzymes or gut microbiota. Indeed, rhamnosides deglycosilation occurs by gut microbiota, suggesting a delayed absorption as compared to the glycosides. The rapid absorption of quercetin glucosides may be assigned to the enzymatic cleavage of cytosolic β-glycosidase or lactase phlorizin hydrolase in the enterocyte. However, the absorption of other flavonoids, such as naringenin and phlorizin, does not seem to be affected by glycosylation. Whereas no differences were found in the identified plasma metabolites, the total concentration was significantly different being higher in a quercetin 3-glucoside meal and non-detectable under quercetin 3-rhamnoside consumption.

Intestinal absorption is also affected by esterification. Indeed, caffeic acid have been described as better absorbed than chlorogenic or quinic acid [142]. However, these studies could be underestimating the yields, since polyphenols pharmacokinetics is still a challenge. Indeed, the cumulative urinary excretion of catechin metabolites was verified to be higher than that of their precursor dietary polyphenol [143,144].

It is important to note that most flavonoids could be absorbed in the stomach or duodenum, since plasma concentration starts to increase after 1–2 h of oral administration. The conservation of a high concentration of polyphenols or polyphenol-derived metabolites in plasma involves a continued ingestion of the polyphenols over time, as previously observed [143]. In a nutritional approach, the whole meal must be considered, as well as the timing of consumption or the fasting/feeding intervals. Moreover, the complex network which leads to polyphenols catabolism, the previously discussed ability of polyphenols to bind with serum proteins, the low concentration of highly different metabolites and other difficulties associated with the cleaning-up of biological samples prior to analysis, indicate the need for further research in order to improve the sensitivity and reliability of the results. Furthermore, targeted metabolomics is the main approach to address this challenge, while the possibility of forming different metabolites remains unexplored. Moreover, the ability of polyphenols to bind with macromolecules could mask the accurate quantification of the absorption rate.

## 4. Challenges on Bioactive Peptide Research

Research in plant-based bioactive peptide faces important challenges. For naturally present bioactive peptides, it is important to consider that, the same as for polyphenols, environmental conditions and agronomical practices can considerably influence bioactive peptide content. Their concentration in crops and ultimately in foods must be considered. These parameters should be further researched and considered to improve the content of bioactive peptides by breeding and by the optimization of growing conditions.

Furthermore, as referred to ahead, a lot of information is present in the literature concerning the bioactivity of peptides from cereals and legumes in vitro. However, it is hard to translate these in vitro experimental observations to humans. As mentioned, the bioactive peptide may be demoted through digestion. Indeed, while, on one side, one of the main concerns in bioactive peptides research is the digestibility of the precursor proteins, on the other hand, additional hydrolysis of the bioactive peptides can lead to the disappearance of their bioactivity.

The in vivo activity-guided fractionation, isolation and identification of protein-digested fractions joined with the in vivo evaluation have been recently used to identify the peptides responsible for the observed bioactivities. Based on these identified “natural sequences”, several peptides have been synthetized. Synthetic peptides usually displayed higher bioactivities. Until now, evidence suggests that promising bioactive peptides can be found and proteomics and transcriptomics have arisen within the last few years, in the search of new sources of bioactive peptides [145].

Indeed, regarding the discovery and validation of bioactive peptides, it has been already suggested that trial-and-error and one-factor-at a time experimentation is largely outdated, having been replaced by systematic scheme of experimentations [146,147]. Some databases have been made to collect what is known about specific bioactive peptides [41], with at least one specific database for plant-based antimicrobial peptides (Phytamp). However, much research is necessary, especially in the field of proteins derived from plant kingdom. The great intricacy and the wide dynamic range of relative peptide abundance in plant-based foods encounter the facilities of current analytical methodologies. Moreover, the few plant protein databases and the high number of homologous proteins and peptides clearly difficult this exploration.

Furthermore, the absorption and transport mechanisms by which bioactive peptides reach the bloodstream and the further effect in the target issues remain underexplored. Pharmacokinetics and bioavailability assays are also needed to understand the mechanisms of action. Additionally, the intake concentration necessary to exert its function and structure/activity relationship studies must be addressed to better understand the real employment by the organism, and also to help in the design of new synthetic bioactive peptides with improved bioactivities.

Most available clinical data on the effects of plant bioactive peptides in humans arises from epidemiological studies. However, to control the effect of bioactive peptides through diet is difficult due to the lack of a proper “placebo diet”, which requires the substitution of the considered “target” cereal or legume. Moreover, the source of peptides is so diverse in a standard diet that it is not enough to replace only a single foodstuff, but it is necessary to alter and severely track the entire diet. The methodology applied to analyze the effect of a bioactive compound from diet is analogous to that typically used from standard clinical trial performed in the study of a drug effect but the number of interactions is much higher. Looking for adequate outcomes, selection criteria, randomization and sample size, diet control and analytical constraints are parameters to track.

From a nutritional approach, it is almost unmanageable to carry on trials to associate the effects of different bioactive peptides in the context of a standard diet if the interactions between the bioactive peptides and other food components are not being considered. Indeed, most bioactive peptides from legumes and cereals are included in proteins and are linked with other bioactive compounds (in particular polyphenols), as is described in the following passages. If the challenge is addressed by using synthetic bioactive peptides like a drug (food supplement), the observed effect should not be larger than the effect expected from nutrients for regulatory reasons, otherwise it must be considered a drug. The ultimate consequence is that the only way to evaluate the effects of plant-based bioactive peptides is to revise the outcome of different diets, rich or poor in bioactive peptides. However, it is important to remark that, even in this case, the interaction between bioactive peptides and the whole diet and host must be considered.

Clinical data concerning the effects of single peptides have been only observed in bioactive peptides from animal origin even if not vegetable ones. Clinical effects and intervention studies of vegetable peptides need to be studied as well.

In summary, bioactive peptides are a nature’s tool kit to protect health, if we are able to control and diminish the risk of unexpected side reactions. However, the lack of information about peptide bioavailability, pharmacokinetics, metabolism and digestion in humans makes it hard to turn the knowledge, obtained from in vitro studies, to humans. Further in vivo research and clinical trials will be required to completely discover the protective/preventive effects of bioactive peptides.

## 5. Conclusions

Among bioactive compounds, health-promoting functional foods, dietary supplements and pharmaceutic formulas containing bioactive peptides or polyphenols are currently of interest for scientific research. The world population is increasing, while the requirement for more non-animal protein-based food is emerging. On the other side, the rise in the incidence of non-communicable diseases promotes the use of natural bioactive compounds able to prevent or control this incidence. These two factors currently drive the research on new and alternative protein sources and product development.

Despite the large scientific knowledge concerning peptides derived from animal sources, the scientific community recently searched to obtain plant-based bioactive peptides. The continued use of plants as food sources or by their therapeutic applications has long been outstanding. A vast array of natural compounds with biological properties occur in plants. This possibility opens a new way to highlight novel functional features adding bioactive peptides health properties with polyphenols health properties.

However, how the interaction between these bioactive compounds present in plant foodstuffs could affect their pharmacokinetics, bioavailability, absorption and metabolism is still poorly known. In this context, this review summarizes the potential implications by which bioactive peptides–polyphenols must be explored in the study of bioactive compounds and health. Further research is needed to understand the true effect of polyphenol-bioactive peptide interactions on the overall health outcomes. 

## Figures and Tables

**Table 1:** Sources of bioactive peptides from the plant kingdom with the relative biological properties.

Food Source	Bioactivity	Reference
**Oat**	Antiglycating agent	[21]
	ACE inhibitory	[22]
	Antihypertensive and Antioxidant	[23]
	Platelet agreggation	[24]
**Barley**	Antiglycating agent	[21]
	Platelet agreggation	[24]
**Wheat**	Immunomodulatory and Antioxidant	[25]
	ACE inhibitory	[26]
**Lupine**	Anti-inflammatory, Preventive-Metabolic disorder (obesity)	[27]
	Reduces ostoeclastogenesis	[28]
**Maize**	Combact Metabolic syndrome	[29]
	Antifungal activity	[30]
**Rice**	ACE inhibitory	[26]
**Rye**	Neuroendocrine cells-Metabolic disorder-Obesity	[31]
	Anti-inflammatory-Mitigating TNF-α-mediated inflammation	[32]
**Spelt**	Antioxidant	[33]
**Walnut**	Antihypertensive and Antioxidant	[23,34]
	Ameliorates Cognitive Impairments and Alters Gut Microbiota-Alzheimer’s disease	[35]
**Pea**	ACE inhibitory	[36]
**Cowpea**	3-hydroxy-3-methyl-glutaryl coenzyme A reductase inhibitory activity	[37]
**Soybean**	Antihypertensive and Antioxidant	[23]
	ACE inhibitory	[26]
**Sesame**	Antihypertensive and Antioxidant	[23]
**Sunflower**	ACE inhibitory	[38]
	Anti-inflammatory (inhibit NFκB)	[39]
**Cocoa**	ACE inhibitory-Antihypertensive	[40]
**Garlic**	Antihypertensive	[41]
**Broccoli**	ACE inhibitory	[41]
**Spinach**	ACE inhibitory	[41]
**Potato**	Antihypertensive	[42]
	Regulate synaptic plasticity and neuronal survival-Alzheimer’s disease	[43]
**Grape**	ACE inhibitory	[41]

ACE, angiotensin I-converting enzyme.
